# Supplementary figures and images for: Identification of novel tetracycline resistance gene tet(X14) and its co-occurrence with tet(X2) in a tigecycline-resistant and colistin-resistant Empedobacter stercoris
Source: Emerg Microbes Infect. 2020 Aug 14;9(1):1843–52. doi: 10.1080/22221751.2020.1803769 (PMC7473080; doi:10.1080/22221751.2020.1803769)

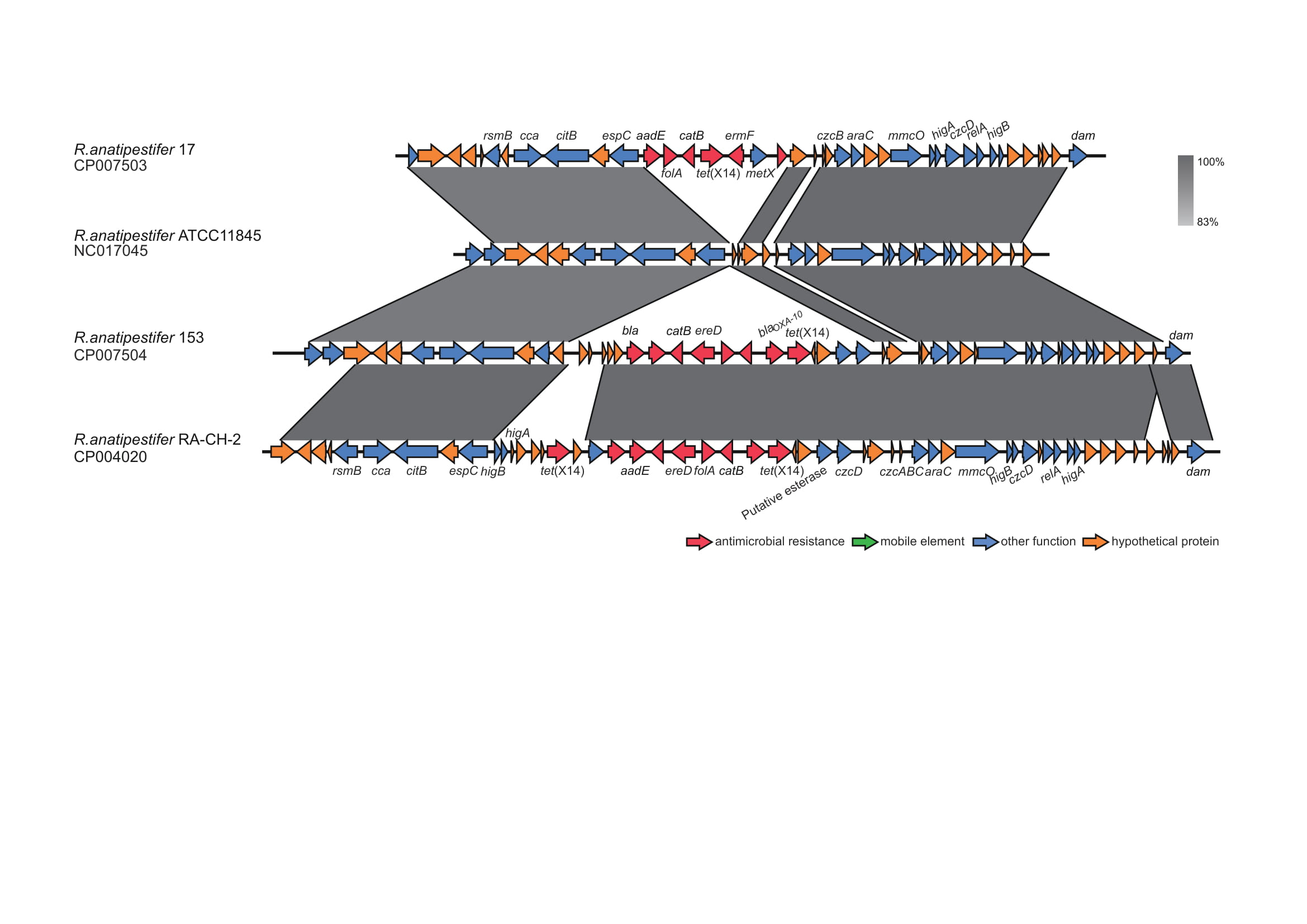

Supplement: Supplementary_Figure_2._Antibiotic_resistance_islands__ARIs__encoding_tet_X14__identified_in_R._anatipestifer-1_final.jpg [file TEMI_A_1803769_SM2573.jpg]

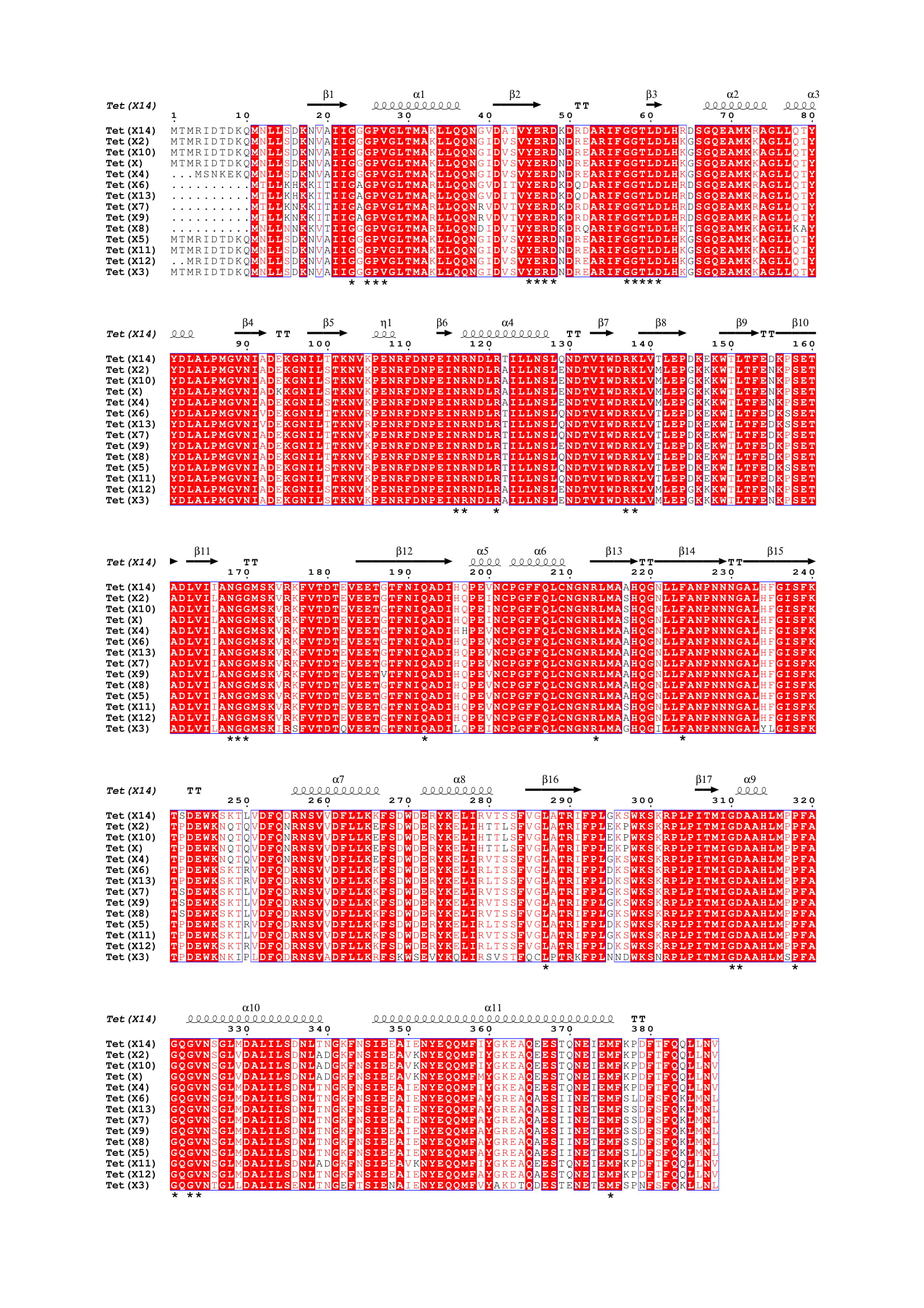

Supplement: Supplementary_Figure_1._The_secondary_structure_of_Tet_X14__and_its_homologs-1_final.jpg [file TEMI_A_1803769_SM2572.jpg]
